# Efficient Removal of Methylene Blue by Bio-Based Sodium Alginate/Lignin Composite Hydrogel Beads

**DOI:** 10.3390/polym14142917

**Published:** 2022-07-19

**Authors:** Tao Chen, Haochen Liu, Jie Gao, Guowen Hu, Yuan Zhao, Xiuqin Tang, Xiaobing Han

**Affiliations:** Hubei Key Laboratory of Radiation Chemistry and Functional Materials, Non-Power Nuclear Technology, Collaborative Innovation Center, Hubei University of Science and Technology, Xianning 437100, China; taochen518@163.com (T.C.); h2250421565@163.com (H.L.); hgwpublic@163.com (G.H.); zhyf308@hbust.edu.cn (Y.Z.); txqtowd20088@126.com (X.T.)

**Keywords:** adsorption, sodium alginate, lignin, microbeads, methylene blue

## Abstract

Dye pollution is a serious issue in current environment protection, and bio-based adsorbents have been receiving much attention in wastewater treatment, due to their low cost, renewable, and environmentally friendly characteristics. Bio-based sodium alginate/lignin composite (SA/Lig) hydrogel beads were fabricated by a facile cross-linking with calcium ion and used for the removal of methylene blue (MB). The obtained SA/Lig microbeads were characterized with SEM, FTIR, and TG, and the effect of lignin content, pH, and temperature on the MB adsorption was investigated. The results indicated that the introduction of aromatic lignin can not only enhance thermal stability but also can improve the adsorption performance. Under optimal conditions, the maximum adsorption capacity (254.3 mg/g) was obtained for the SA/Lig-20% beads, with a removal efficiency of 84.8%. The adsorption process for MB is endothermic, and the rate-limiting step is chemical adsorption. The removal efficiency is higher than 90% after five cycles, revealing that the prepared beads show good regeneration ability.

## 1. Introduction

Nowadays, synthetic dyes have been widely used in the textile industries, leading to serious pollution of water resources [1,2]. Among these dyes, methylene blue (MB) is a commonly used cationic dye, which is more damaging to the environment and humans, as it can be easily combined with negatively charged cell membranes [3,4]. The treatment of MB in wastewater is an urgent issue, and many methods have been developed for the removal of MB. Compared with other methods, adsorption is superior in efficiency and economically [5,6].

To meet the requirements of sustainable development, the renewable, ecofriendly adsorbents originating from biomass were developed for the removal of MB in wastewater. Bio-based lignin-chitosan pellets were fabricated by the Albadarin group [7], and used for the removal of MB, where the maximum adsorption capacity is 36.25 mg/g and the adsorption process can be explained by the pseudo-second-order-model. Marrakchi reported the adsorption of MB with a cross-linked chitosan/sepiolite composite film [8], where the maximum monolayer adsorption capacity is 40.986 mg/g, which was obtained for the CS50SP50 under optimal conditions. Bio-based sodium alginate/soybean extract beads reinforced with hemp hurd and halloysite were reported by Viscusi for the adsorption of MB [9], and the adsorption capacity reached 49 mg/g when adding 35 wt% halloysite. The results of kinetic fitting revealed that the adsorption was based on the chemisorption mechanism. Based on the magnetic separation technology, functional bead adsorbents consisting of iron oxide, activated carbon, and sodium alginate were also reported for the removal of MB [10], and the results showed a maximum adsorption capacity of 222.3 mg/g with a high initial concentration of 700 mg/L. Recently, covalently cross-linked alginate/montmorillonite composite cryogel with high porosity was reported for the adsorption of MB [11], and the cryogel exhibited effective dye adsorption capacity, where the maximum adsorption capacity against MB was 559.94 mg/g, determined using linear regression of the Langmuir model with an initial concentration of 1000 mg/L.

On the other hand, the intermolecular interaction between the adsorbents and the dye is also an important factor in the design and application [12]. The commercial dyes are aromatic molecules with a negative or positive charge and possess nitrogen or oxygen groups. Based on these features of dyes, electrostatic attraction, π-π stacking, and a hydrogen bond can be designed in the construction of adsorbents. Due to its ionic characteristic, the electrostatic attraction was first taken into account in the adsorbents’ design, thus the bio-based positive chitosan and negative cellulose alginate are the most used polymer matrices [7,8,9,10,11]. As the dyes are aromatic molecules, bio-based aromatic tannin- and lignin-based adsorbents have also been developed based on the π-π stacking interaction [13,14,15]. In addition, the hydrogen bond is another intermolecular interaction involved in the construction of bio-based adsorbents, which can be easily formed between the nitrogen- or oxygen-containing groups in dyes and biomass.

As mentioned above, for the removal of aromatic methylene blue (MB) with nitrogen, bio-based aromatic lignin (Lig) with a hydroxyl group and negative sodium alginate (SA) were used for the adsorbent construction. Bead-type and millimeter-sized SA/Lig composite hydrogel beads were fabricated by a facile cross-linking with calcium ion, and the obtained SA/Lig beads were characterized and used for the removal of MB. The effects of the experimental, thermodynamic, kinetic, and regeneration parameters were also investigated.

## 2. Materials and Methods

### 2.1. Materials

Sodium alginate (SA, 200 mPa·s), Lignin (Lig, 96%, Mw = 10,000 g/mol), CaCl_2_, and methylene blue (MB, 98%) were purchased from Huaweriruike chemical Co. Ltd. (Beijing, China). Distilled water was used for MB solution preparation.

### 2.2. Preparation of SA/Lig Composite Hydrogel Beads

The sodium alginate/lignin composite hydrogel beads were prepared as follows [5,6]: Firstly, an amount of lignin was dispersed into 50 mL of water under sonication. Then, 1.0 g of sodium alginate was added to the dispersion at 60 °C under stirring until complete dissolution (the weight percentage of lignin to sodium alginate was 0, 10, 20, 40, 80 wt%). At room temperature, without stirring, the above mixture was added dropwise into a 5 wt% CaCl_2_ solution, and the cross-linking reaction was maintained for 24 h. SA/Lig composite beads were obtained with filtration, and then washed with distilled water.

### 2.3. Characterization of SA/Lig Composite Beads

The surface morphology of the obtained composite beads was determined by scanning electron microscopy (SEM, VEGA-3, Tescan, Czech Republic). The chemical structure of the composites was revealed with Fourier transform infrared (FTIR, Avatar 360 Nicolet instrument). The thermogravimetry (TG) analysis was conducted via a TG 209F3 instrument (NETZSCH Scientific Instruments Ltd., Shanghai, China) under an N_2_ atmosphere. The absorbance of the MB solution was determined with an S 3100 spectrophotometer (Mapada Instruments Co., Ltd., Shanghai, China), and the standard curve (*A* = 0.182*C* − 0.017, and *A* and *C* are the absorbance and concentration of MB, respectively) was obtained with the determination of MB absorbance at 664 nm.

### 2.4. Removal of Methylene Blue

The adsorption of MB from the aqueous solution onto SA/Lig beads was conducted by the batch procedure. Then, 20 mg of dried SA/Lig adsorbents were soaked in 30 mL of the MB solution, then the solution was placed in an isothermal water bath shaker at 150 rpm. The pH of the solution was adjusted with 0.1 M NaOH and an HCl solution. The concentration of MB was measured using UV-vis spectroscopy at 664 nm [15]. The adsorption capacity *Q* (mg/g) and removal efficiency *R* (%) was calculated as follows:

*Q* = (*C_i_* − *C_t_*)*V/m* (1)

*R* = (*C_i_* − *C_t_*) × 100/*C*_0_ (2)

*C_i_* (mg/L) and *C_t_* (mg/L) refer to the MB concentration before and after adsorption, W (g) is the dosage of SA/Lig adsorbents, and *V* (L) is the volume of the aqueous solution.

## 3. Results and Discussion

The goal of this work was to reveal the morphology, chemical structure, and thermal stability of the obtained SA/Lig beads, as well as the influence of lignin content, pH, and temperature on the MB adsorption performance. In addition, the thermodynamics, kinetics, and regeneration were also investigated to reveal the adsorption mechanism, thermic effect, and recyclability.

### 3.1. Fabrication and Characterization of SA/Lig Composite Beads

#### 3.1.1. Surface Morphology

**Figure 1:**
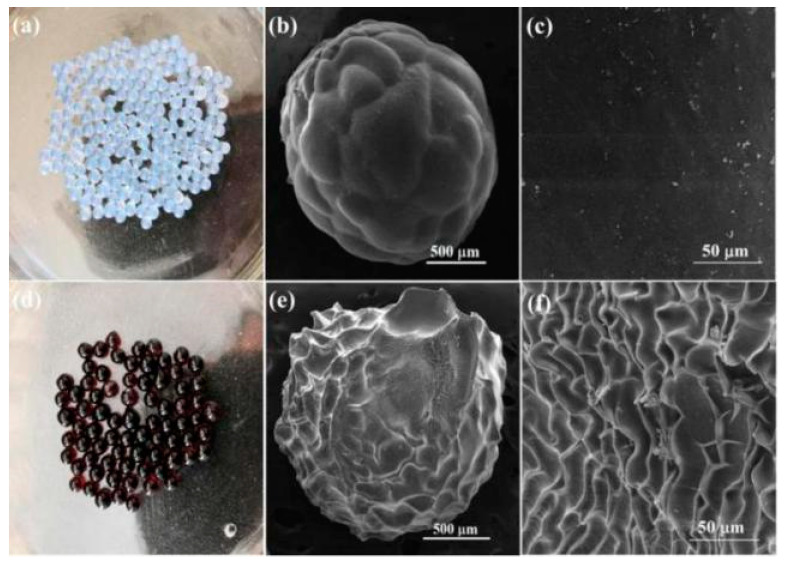
Photos (**a**) and SEM images (**b**) ×100, (**c**) ×1000 of pure SA beads, photos (**d**) and SEM images (**e**) ×100, (**f**) ×1000 of SA/Lig-20% beads.

The pictures and surface morphology of pure and SA beads and SA/Lig-20% beads are shown in Figure 1. The obtained composite beads are spherical in shape and show uniform size distribution. The pure SA beads show a colorless and transparent appearance with a diameter of approximately 2 mm, while the color changed to red-brown with a diameter of approximately 1.5 mm for SA/Lig-20% beads. The pure SA beads present a relatively smooth surface similar to the chitosan/gelatin/graphene beads [16], while the SA/Lig beads show a wrinkled morphology with an enhanced surface area, similar to the lignin-containing cellulose nanocrystals/sodium alginate beads [5]. This illustrates that the incorporation of aromatic lignin into the sodium alginate enhances the surface area of the beads, which will benefit the adsorption of MB. These adsorbents are millimeter-sized beads, similar to the commercial macroporous adsorption resin, which will benefit the application in industry [17].

#### 3.1.2. Composition Analysis

**Figure 2:**
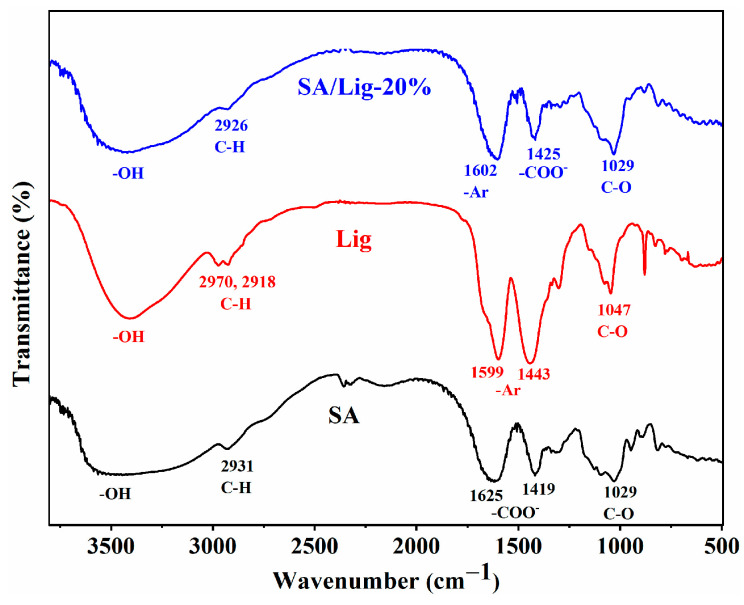
FTIR spectra of SA, Lig, SA/Lig-20% beads.

The FTIR spectra of SA, Lig, and SA/Lig-20% beads are shown in Figure 2. In the spectra of SA, the peak at 1029 cm^−1^ is assigned to the stretching of C-O, while the peaks at 1419 and 1625 cm^−1^ are attributed to the symmetric and asymmetric stretching of carboxylate groups [18,19]. For the spectra of Lig, the broad band at 3415 cm^−1^ is ascribed to the -OH group, while the peaks at 1599 and 1443 cm^−1^ refer to the C-C stretching vibration for the aromatic skeleton [5], which is beneficial for the adsorption of MB through the formation of the π-π stacking interaction. For the SA/Lig-20% beads, all the peaks of SA and Lig can be observed, and the symmetric stretching of carboxylate shifts from 1419 to 1425 cm^−1^, which is consistent with the lignin/sodium alginate composite cross-linked via calcium ion [5,6], demonstrating the formation of SA/Lig composite beads.

#### 3.1.3. Thermal Stability

**Figure 3:**
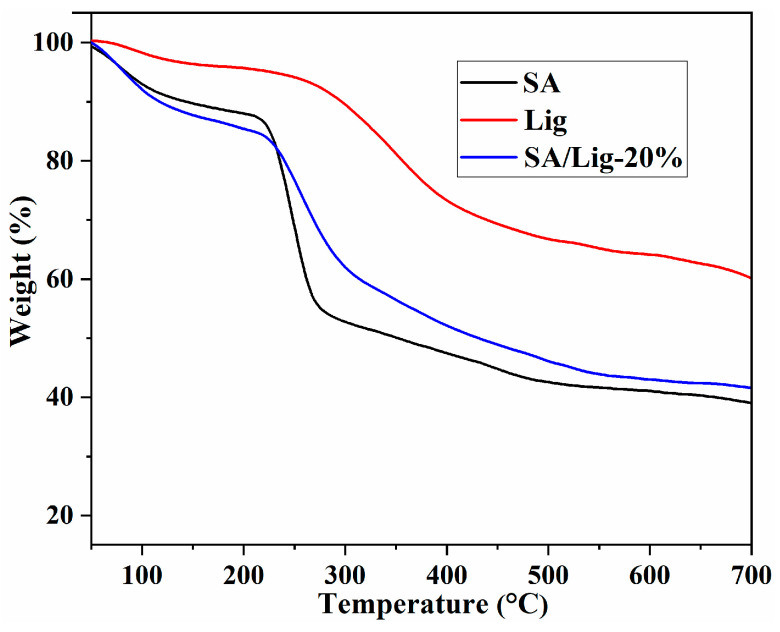
TG spectra of SA, Lig, SA/Lig-20% beads.

The TG curves of SA, Lig, and SA/Lig-20% beads are shown in Figure 3. Lig shows the highest thermal stability with three degradation steps: The weight loss occurred below 150 °C owing to the loss of the absorbed water; the obvious degradation process at approximately 250 to 400 °C can be associated with the decomposition of oxygen-containing groups and cleaving of C-C bonds; and the last weight loss after 400 °C might be attributed to the degradation of the aromatic structure [20,21,22]. For the TG curves of SA, the weight loss of absorbed water is approximately 8% before 120 °C. In the second stage, the weight loss of approximately 6% between 150 and 230 °C can be ascribed to the decarboxylation of SA. The weight loss between 230 and 450 °C is due to the decomposition of the aminopropyl groups and bisaldehyde, and the organic species have been completely decomposed after 450 °C [10]. For the curve of SA/Lig-20% beads, degradation behavior similar to SA and improved thermal stability were observed for the composite beads, which can be attributed to the incorporation of the rigid structure of aromatic Lig molecules and the calcium ion cross-linking [10,13].

### 3.2. Removal of MB with SA/Lig Beads

#### 3.2.1. Effect of Lignin Content

**Figure 4:**
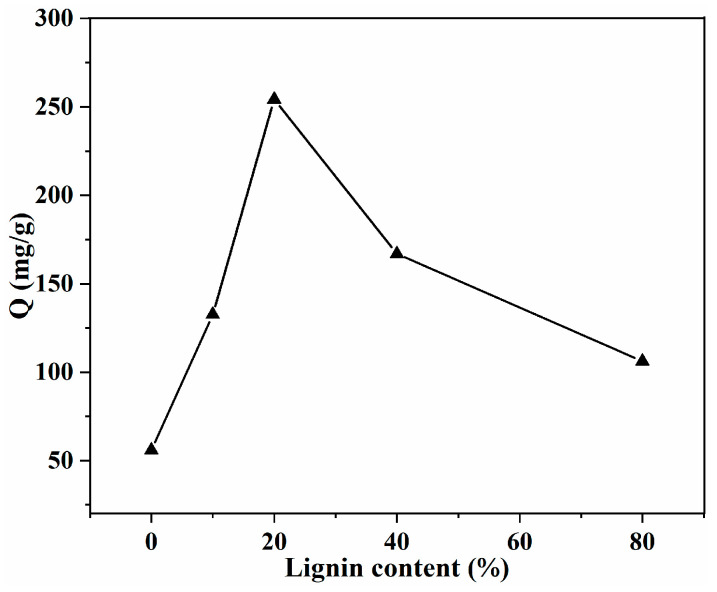
Effect of Lignin content on the adsorption capacity (*m* = 20 mg, *C*_0_ = 200 mg/L, *V* = 30 mL, pH = 7, *T* = 25 °C, *s* = 150 r/min, *t* = 24 h).

The adsorption capacity of MB with different SA/Lig beads is shown in Figure 4. With the incorporation of aromatic lignin, the adsorption capacity of the composite beads clearly was enhanced. The maximum adsorption capacity (254.3 mg/g) was observed for the SA/Lig-20% adsorbent, with a removal rate of 84.8%. This is much higher than that of SA beads, which can be attributed to the formation of intermolecular interaction such as π-π stacking and a hydrogen bond between the lignin component and the MB molecules, similar to the adsorption of Congo red onto graphene oxide/chitosan pellets [23,24]. When the content of lignin exceeded 20%, the adsorption capacity decreased, which can be ascribed to the hindered effect of excessive rigid lignin for the diffusion of MB molecules, which is consistent with the results of the lignin/chitosan beads [13] and chitosan/graphene composite spheres [25]. As the highest adsorption capacity was obtained for the SA/Lig-20% beads, these adsorbents were thus chosen for further investigation.

#### 3.2.2. Effect of pH Value

**Figure 5:**
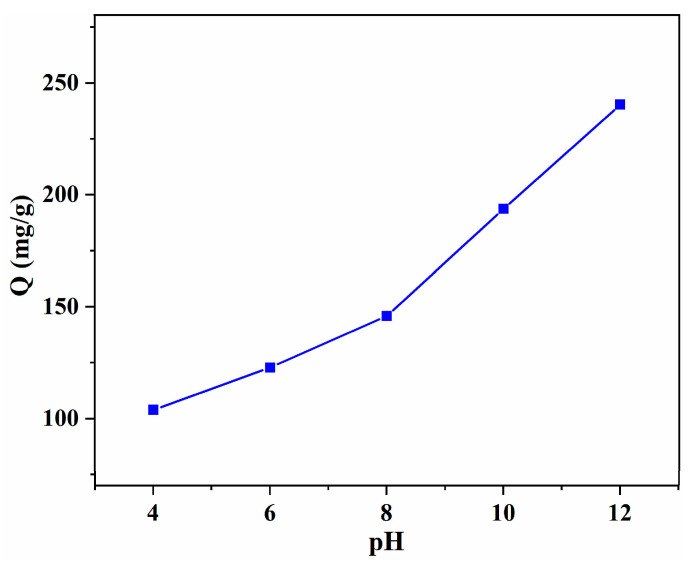
Effect of the pH value on the adsorption capacity with SA/Lig-20% beads (*m* = 20 mg, *C*_0_ = 200 mg/L, *V* = 30 mL, *T* = 25 °C, *s* = 150r/min, *t* = 4 h).

As the pH value is one of the most important parameters that influence the adsorption performance, the effect of the solution pH (4–12) on the adsorption capacity was investigated with SA/Lig-20% beads. As shown in Figure 5, the adsorption capacity shows a high dependence on the pH value, revealing that electrostatic attraction plays an important role in the adsorption of ionic MB. The highest adsorption capacity (240.3 mg/g) was obtained at a pH of 12, with a removal efficiency of 80.1%. This can be attributed to the disadvantage of the high H^+^ concentration, which has competitive adsorption with positive MB, and a similar phenomenon was observed in the methylene blue and Congo red removal [13,14,15]. Taking the protection of the environment and equipment into account, adsorption at a higher pH value (>12) was not conducted.

### 3.3. Adsorption Thermodynamic

**Figure 6:**
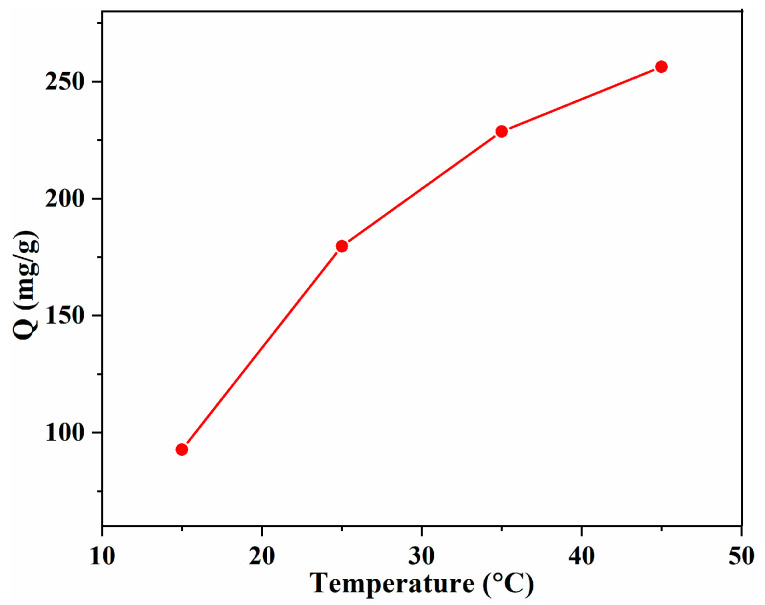
Effect of the temperature on the adsorption capacity with SA/Lig-20% beads (*m* = 20 mg, *C*_0_ = 200 mg/L, pH = 12, *s* = 150 r/min, t = 4 h).

The temperature is another significant parameter that can reveal the thermic effect of the adsorption process. The effect of the temperature on the adsorption capacity of the SA/Lig-20% beads is shown in Figure 6. As the temperature increased, the adsorption capacity increased gradually, revealing that adsorption is an endothermic process. This can be ascribed to the increase in the mobility of MB molecules and the number of active sites of the composite adsorbents, which is similar to the adsorption of anionic dye with palm kernel fibers [26]. The maximum adsorption capacity (256.3 mg/g) was observed at 45 °C, and adsorption at higher temperatures was not conducted as it required more energy.

The thermodynamic parameters including *K*^0^, Δ*G*^0^, Δ*H*^0^, and eΔ*S*^0^ can be calculated by the following equations [13,27]:

*lnK*^0^ = *ln*(*Q_e_*/*C_e_*) (3)

Δ*G*^0^ = −*RT ln K*^0^ (4)

*Rln K*^0^ = −Δ*H*^0^/*T* + Δ*S*^0^ (5)

where *Q_e_* is the adsorption capacity, *C_e_* is the equilibrium concentration, *R* is the universal gas constant (8.314 J/mol·K), and *T* is the temperature in Kelvin.

The Δ*H*^0^ and Δ*S*^0^ are calculated from the linear plot of ln*K*^0^ versus 1/*T*. The calculated parameters are listed in Table 1. With the increase in the temperature, the Δ*G*^0^ decreased dramatically, revealing that an enhanced temperature is favorable for the adsorption, which is consistent with the results mentioned above. When the temperature is 288 K, the value of Δ*G*^0^ is positive, revealing that adsorption cannot proceed spontaneously [15]. With a further increase in the temperature, the value of Δ*G*^0^ changes to negative, indicating adsorption can proceed spontaneously at high temperatures. Δ*H*^0^ and Δ*S*^0^ calculated from the slope and intercept are 64.19 kJ/mol and 0.22 kJ/mol·K, respectively, and the positive value of Δ*H*^0^ demonstrated an endothermic feature of the MB adsorption.

### 3.4. Adsorption Kinetic

**Figure 7:**
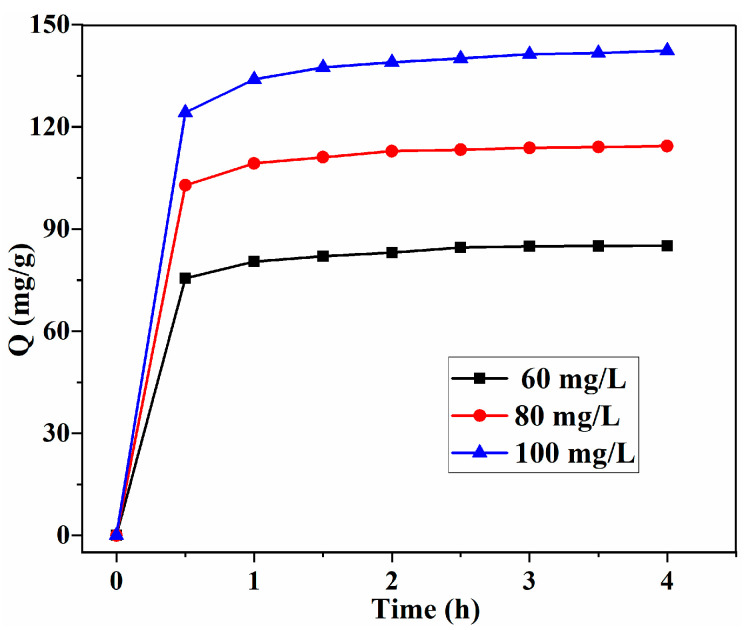
Effect of initial concentration and contact time on the adsorption capacity with SA/Lig-20% beads (*m* = 20 mg, *V* = 200 mL, pH = 12, T = 45 °C, *s* = 150r/min).

The effect of the initial concentration and contact time on the adsorption of MB is shown in Figure 7. As the initial concentration increased, the equilibrium concentration increased, indicating that MB adsorption is dependent on the initial concentration [13]. It will take a long time to diffuse into the adsorbents’ matrix for MB molecules after the saturation adsorption, which is similar to the removal of MB with bio-based chitosan microspheres grafted with sodium polystyrene sulfonate [15].

The adsorption kinetics can reveal the adsorption mechanism and efficiency and determine the application of the fabricated adsorbents. To provide deeper insight into the adsorption process and mechanism of MB adsorption onto SA/Lig beads, pseudo-first-order and pseudo-second-order kinetic models were used to evaluate the adsorption kinetic (Equations (6) and (7)) [28,29,30,31,32,33].

Pseudo-first-order:

(6) $$\log(q_{e}-q_{t})=\log q_{e}-k_{1}t$$

Pseudo-second-order:

(7) $$\frac{t}{q_{t}}=\frac{1}{k_{2}q_{e}^{2}}+\frac{t}{q_{e}}$$

where *q_e_* and *q_e_* are the adsorption capacity at equilibrium and time *t*, respectively, and *k*_1_ and *k*_2_ are the rate constants for the two models.

**Figure 8:**
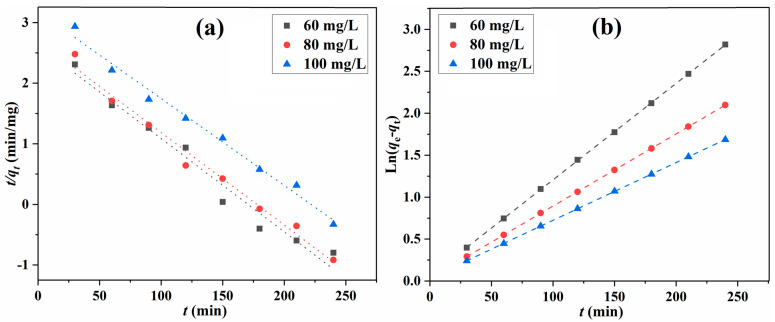
Fitting of pseudo-first-order (**a**) and pseudo-second-order kinetic (**b**) models for the adsorption process.

The fitting curves of the adsorption with different kinetic models are shown in Figure 8, and the calculated parameters are summarized in Table 2. The experimental value is very close to that calculated for the pseudo-second-order model, with the equilibrium adsorption capacity at different initial concentrations. The correlation coefficient R^2^ (0.9999) shows that this model fits the experimental data better than that of the pseudo-first-order model, demonstrating that chemical adsorption is the rate-limiting step for the MB molecules adsorption [13,15].

### 3.5. Regeneration Performance

**Figure 9:**
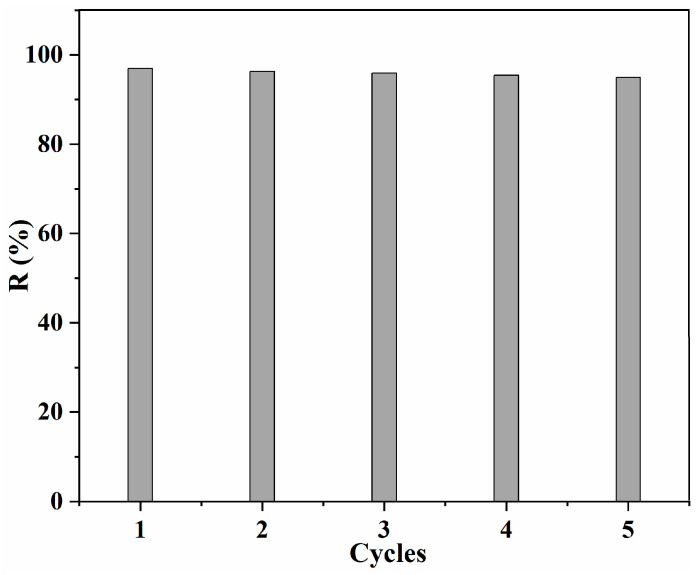
Effect of recycling times on the adsorption capacity Q (mg/g) with 40%Tan/SA beads (*m* = 20 mg, *C*_0_ = 10 mg/L, *V* = 30 mL, pH = 12, *T* = 45 °C, *s* = 150 r/min, *t* = 4 h).

The regeneration performance is also an important factor for the adsorbents, therefore the regeneration behavior of the obtained SA/Lig beads was evaluated with the desorption experiment. The desorption of MB from SA/Lig-20% beads was realized with a 1 M HCl aqueous solution in an incubator shaker, washed with distilled water. As shown in Figure 9, the removal efficiency decreased slightly but was still higher than 90% after five cycles, at which point the adsorption capacity decreased from 14.55 mg/g to 13.80 mg/g, revealing that the obtained SA/Lig beads have a good regeneration ability [13,15]. This is much better than the MB removal with Kaolin [32], with a removal efficiency that decreased from 56% to 23% after four cycles.

### 3.6. Adsorption Performance Comparison

The maximum adsorption capacity, removal efficiency, and regeneration of the obtained SA/Lig beads were compared with bio-based materials in the literature [34,35]. As shown in Table 3, there are almost no regeneration data for the reported adsorbents [7,8,9,10,11]. Though some of the adsorbents show a much high adsorption capacity at a high initial concentration, the removal efficiency is relatively low. The SA/Lig beads show a relatively high adsorption capacity and removal efficiency compared to most similar bio-based adsorbents, and the improved adsorption performance can be attributed to the combination of aromatic lignin with SA [13,14].

## 4. Conclusions

In summary, bio-based sodium alginate/lignin composite hydrogel beads were fabricated by facile cross-linking with calcium ions and were used for the efficient removal of methylene blue. The incorporation of aromatic lignin into sodium alginate not only improved the thermal stability of the obtained adsorbents but also enhanced the adsorption performance. The maximum adsorption capacity of 254.3 mg/g (removal efficiency of 84.8%) was obtained for SA/Lig-20%, under the optimal conditions of pH = 12, T = 45 °C. The calculated thermodynamic and kinetic parameters revealed that adsorption is an endothermic process, and chemical adsorption is the rate-limiting step. In addition, the obtained adsorbents show a good regeneration ability. The prepared SA/Lig composite beads have promising applications for the removal of cationic dyes from wastewater, and future work will focus on the selectivity, scale-up, and continuous column adsorption of these adsorbents.

## Figures and Tables

**Table 1:** Thermodynamic parameters for the adsorption of MB on SA/Lig-20% beads.

Temperature (K)	ln*K*^0^	Δ*G*^0^ (kJ/mol)	Δ*H*^0^ (kJ/mol)	Δ*S*^0^ (kJ/mol·k)
288	−0.805	1.93	64.19	0.22
298	0.398	−0.99	-	-
308	1.166	2.98	-	-
318	1.77	4.68	-	-

**Table 2:** Kinetic parameters of two models for MB adsorption.

Kinetic Models	Coefficients	60 mg/L	80 mg/L	100 mg/L
Pseudo-first-order	*q*_e,cal_ (mg/g)	13.82	15.06	24.05
	*k*_1_ (min^−1^)	0.0154	0.0163	0.0144
	*R* ^2^	0.9711	0.9846	0.9871
Pseudo-second-order	*q*_e,cal_ (mg/g)	87.03	116.28	145.35
	*k*_2_ (×10^−3^) (g/mg min)	2.34	2.21	1.32
	*R* ^2^	0.9999	0.9999	0.9999

**Table 3:** Comparison of the MB removal performance with similar adsorbents.

Adsorbent	Experimental Conditions (*C*_0_/(mg/L), pH, T/°C, t/h)	*Q_max_* (mg/g)	Removal (%)	Ref.
SA/Lig	200, 12, 45, 24	254.3	84.8	This work
Lig/CS	82, 7, 20, 40	36.25	88.4	[7]
CS/SP	300, 9, 30, 24	40.986	27.3	[8]
SA/SB/HNT	100, 7, 25, 24	49	94	[9]
Fe_3_O_4_/AC/SA	700, 7, 25, 4	222.3	31.8	[10]
SA/MMT	1000, 7, 25, 24	559.94	56	[11]

## Data Availability

Not applicable.
